# Magnetic Persimmon Leaf Composite: Preparation and Application in Magnetic Solid-Phase Extraction of Pesticides in Water Samples

**DOI:** 10.3390/molecules29010045

**Published:** 2023-12-20

**Authors:** Yuyue Zang, Na Hang, Jiale Sui, Senlin Duan, Wanning Zhao, Jing Tao, Songqing Li

**Affiliations:** Beijing Key Laboratory for Forest Pest Control, College of Forestry, Beijing Forestry University, No. 35 Qinghua East Road, Beijing 100083, China; zangyuyue@126.com (Y.Z.); 15044764521@163.com (N.H.); suijiale1019@163.com (J.S.); dslm0807@bjfu.edu.cn (S.D.); furongzhuoqiuyu@bjfu.edu.cn (W.Z.); taojing1029@hotmail.com (J.T.)

**Keywords:** biomass adsorbent, Fe_3_O_4_/persimmon leaf magnetic composite, magnetic solid-phase extraction, pesticides, water

## Abstract

In recent years, the utilization of biomass materials for the removal and detection of water pollutants has garnered considerable attention. This study introduces, for the first time, the preparation of Fe_3_O_4_/persimmon leaf magnetic biomass composites. The magnetic composites were employed in a magnetic solid-phase extraction method, coupled with gas chromatography-electron capture detection (GC-ECD), for the analysis of four pesticides (trifluralin, triadimefon, permethrin, and fenvalerate) in environmental water samples. The innovative magnetic persimmon leaf composites were synthesized by in situ generation of Fe_3_O_4_ nanoparticles through coprecipitation and loaded onto persimmon leaves. These composites exhibit superparamagnetism with a saturation magnetization of 12.8 emu g^−1^, facilitating rapid phase separation using a magnetic field and reducing the extraction time to 10 min. Desorption can be achieved within 30 s by aspirating 20 times, eliminating the need for time-consuming and labor-intensive experimental steps like filtration and centrifugation. The specific surface area of the magnetic composite adsorbent increased from 1.3279 m^2^ g^−1^ for the original persimmon leaf to 5.4688 m^2^ g^−1^. The abundant hydroxyl and carboxyl groups on the composites provide ample adsorption sites, resulting in adsorption capacities ranging from 55.056 mg g^−1^ to 73.095 mg g^−1^ for the studied pesticides. The composites exhibited extraction recoveries ranging from 80% to 90% for the studied pesticides. Compared to certain previously reported MSPE methods, this approach achieves equivalent or higher extraction recoveries in a shorter operation time, demonstrating enhanced efficiency and convenience. Good linearity of the target analytes was obtained within the range of 0.75–1500 μg L^−1^, with a determination of coefficient (*R*^2^) greater than 0.999. These findings contribute to the use of magnetic persimmon leaf biomass materials as effective and environmentally friendly adsorbents for pollutant determination in water samples.

## 1. Introduction

Chemical control is one of the primary measures for managing plant diseases and pests. Nitroaniline herbicides, triazole fungicides, and pyrethroid insecticides are commonly employed pesticides in forestry, demonstrating significant efficacy in controlling various prevalent pests and diseases in the forest ecosystem [1,2,3]. After pesticides are sprayed, they inevitably enter the environment, leading to environmental pollution and the extermination of beneficial organisms. In various environmental media, water bodies are particularly susceptible to pesticide residue pollution. Pesticide residues have strong mobility and enrichment capabilities in flowing bodies of water, including streams, lakes, ponds, and groundwater [4,5]. Pesticides such as imidacloprid, chlorpyrifos, and cypermethrin are not only highly toxic to aquatic organisms like fish and shrimp but also pose a significant threat to pollinators like bees and silkworms [6]. This can result in long-term adverse effects on the environment [7]. Moreover, pesticides entering the environment can undergo bioaccumulation through the food chain, eventually posing health risks to humans. The World Health Organization (WHO) has established maximum acceptable concentrations (MAC) for chemical pollutants in drinking water. For instance, the MAC for triadimefon is set at 20 μg L^−1^, and the MAC for permethrin is derived to be 300 μg L^−1^ [8]. Therefore, conducting monitoring and detection of pesticides in water bodies is of paramount importance for green conservation and prevention.

Sample pretreatment plays a critical role in the entire process of pesticide residue analysis [9,10]. Solid-phase extraction (SPE) is one of the most widely used sample pretreatment methods and is capable of separating target analytes from samples [11,12]. Nonetheless, it comes with inherent drawbacks, including labor-intensive and time-consuming procedures, limitations in sample flow rates, and the risk of adsorbent pores getting clogged [13,14]. Therefore, new methods are continuously being developed to rapidly separate analytes from samples. Solid-phase microextraction (SPME) is a solvent-free sample preparation technique that combines sampling, separation, and enrichment into a single step [15,16]. This method was introduced in 1990 to overcome the major drawbacks of conventional SPE, including complexity in automation and instrument design [17]. This method eliminates the need for organic solvents and significantly shortens the analysis time [18]. SPME has gained wide attention due to its advantages, such as high sensitivity, solvent-free methods, and simplicity of operation. Many successful separations and enrichments of target analytes have been performed using this method [19,20,21,22]. 

Magnetic solid-phase extraction (MSPE) is a novel technique composed of magnetic inorganic materials and non-magnetic adsorbents. It is based on traditional SPE and SPME technology [23]. Since its introduction in 1999 by Safarikova [24], it has garnered significant interest and has become one of the main branches of sample preparation methods in recent years [25,26]. The use of magnetic adsorbents enables rapid sample separation under an external magnetic field, streamlining and expediting the operational steps [27,28,29]. MSPE retains the advantages of SPE/SPME and offers several benefits: (1) it has excellent magnetic adsorption capacity, resulting in a short extraction time and significant time savings; (2) selected magnetic adsorbents are highly selective; (3) it enhances the enrichment effect on target compounds, enabling automation of the entire process. These advantages indicate the significant potential of MSPE technology for trace-level analysis of various compounds. Additionally, the adsorption capacity of the magnetic material mainly depends on the nature of the adsorbent material, and the type of adsorbent directly influences the extraction process. Magnetic materials are widely applied in sample preparation due to their excellent extraction performance [30,31,32,33]. For instance, Fe_3_O_4_@C-NFs composites were synthesized through a one-pot synthesis method, achieving efficient separation of tetracycline (TC) in less than 5 min with the assistance of ultra-high performance liquid chromatography (UHPLC) [34]. Another illustration involves the utilization of magnetic molecularly imprinted polymers (MMIP) for the absorption of bisphenol A (BPA) from wastewater samples. The excellent selectivity of MMIP for BPA renders it effective as an MSPE adsorbent in conjunction with spectroscopic instruments [35]. Furthermore, in another study, magnetic cyclodextrins cross-linked with tetrafluoroterephthalonitrile (Fe_3_O_4_@TFN-CDPs) were developed for detecting pesticides in medicinal plants. The study revealed limits of detection (LOD) ranging from 0.011 to 0.106 μg kg^−1^ for the target pesticides [36]. Hence, a key focus in MSPE research is developing highly efficient magnetic adsorbents with robust properties.

Biomass can be broadly defined as any organic material derived from renewable biological sources [37]. Waste biomass possesses irregular surfaces, varying pore sizes, and active functional groups and is abundant in nature [38]. Converting waste, such as biomass, into materials suitable for environmental applications is a green and eco-friendly waste management approach. The use of adsorbent materials developed from abundant plant residues has the potential to rationalize forest resources [39]. In recent years, there has been increasing attention towards waste biomass, mainly composed of cellulose, hemicellulose, lignin, pectin, or tannin. These materials are rich in functional groups like -COOH, -OH, -C=O, -OCH_3_, -NH_2_, -CONH_2_, etc. [40]. Using waste biomass as adsorbents has the advantages of wide availability, short growth cycles, and minimal secondary pollution to the environment. They also offer abundant functional groups and adsorption sites, making them potential biomass adsorbents [41]. Preparing functional materials from plant samples aligns with the principles of green chemistry, organic agriculture, and sustainable agriculture and has gained increasing attention [42,43,44]. 

Persimmon (*Diospyros kaki*) belongs to the family *Ebenaceae*. It is widely distributed in East Asian countries such as China, Japan, and the Republic of Korea, with a cultivation history of several hundred years [45,46]. Persimmon fruits are typically harvested from September to November, leaving a significant amount of persimmon leaves after the harvest [47]. Persimmon leaves are a natural material rich in hydroxyl groups, making them highly suitable for adsorption. Being a byproduct of persimmon trees, they represent a natural adsorbent [48]. From a material cost and environmental perspective, it seems promising to develop persimmon leaves as an adsorbent for various applications. Previous research by Yu and Choi [49] involved the preparation of hybrid beads by mixing chitosan with persimmon leaves. These hybrid beads were structurally capable of adsorbing heavy metals easily due to their carboxyl and carbonyl functional groups. This method was used for the removal of Pb (II) and Cd (II) from aqueous solutions. 

The utilization of persimmon leaves as a magnetic biomass adsorbent, coupled with MSPE technology, holds significant promise for the effective adsorption of pesticides in environmental water. Firstly, the abundance of hydroxyl groups in persimmon leaves provides numerous binding sites, enhancing their compatibility with Fe_3_O_4_. On the other hand, the inclusion of Fe_3_O_4_ nanoparticles elevates the specific surface area and adsorption sites of the magnetic composites. Additionally, the hydroxyl and carboxyl groups in persimmon leaves facilitate intermolecular hydrogen bonds with analytes containing carbonyl and ester groups, while the phenyl structure in persimmon leaves readily forms π-π interactions with pesticides featuring phenyl or heterocyclic groups, thereby bolstering the adsorption process. Consequently, magnetic persimmon leaf composites were synthesized in this study. Waste agricultural and forestry persimmon leaves were utilized as an adsorbent in MSPE, developing a fast and convenient MSPE method. This method was applied in combination with gas chromatography-electron capture detection (GC-ECD) for the detection of four pesticides in environmental water samples. The influence factors on the extraction performance were optimized, and the optimized experimental conditions were determined. The method was evaluated using parameters such as the linear range, LOD, limit of quantitation (LOQ), and other analytical performance parameters. In addition, the reusability of the magnetic persimmon leaf composites was investigated. 

## 2. Results and Discussion

### 2.1. Characterization of Fe_3_O_4_/Persimmon Leaf Magnetic Composite

#### 2.1.1. Fourier Transform Infrared Spectroscopy (FTIR) Analysis

**Figure 1:**
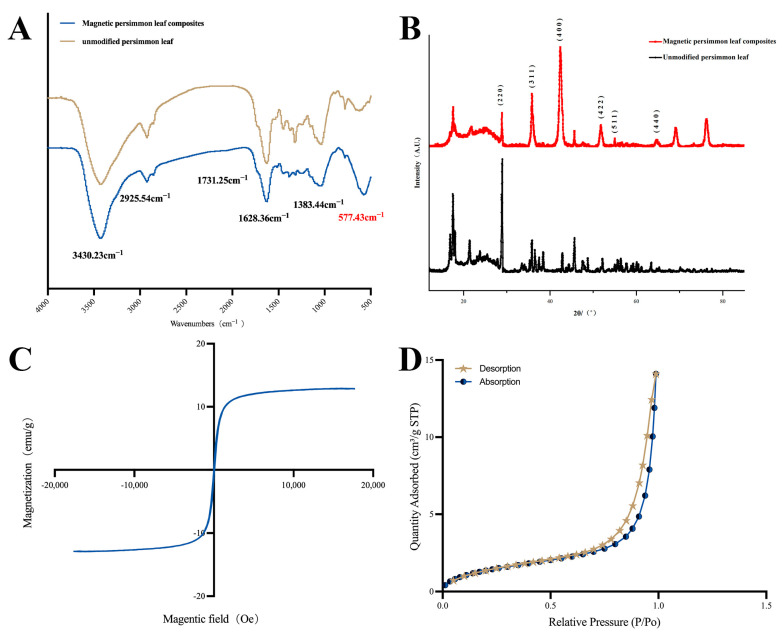
(**A**) Fourier transform infrared spectrum of magnetic persimmon leaf composites and unmodified persimmon leaf; (**B**) X-ray diffraction plot of magnetic persimmon leaf composites and unmodified persimmon leaf; (**C**) Vibrating sample magnetometer of magnetic persimmon leaf composites; (**D**) N_2_ adsorption-desorption isotherms of magnetic persimmon leaf composites.

FTIR was employed in the range of 4000–400 cm^−1^ to investigate the structural characteristics of functional groups on the material surfaces. Figure 1A displays the infrared spectra of both unmodified persimmon leaf particles and magnetic persimmon leaf composites. For unmodified persimmon leaf particles, the peak at 3430.23 cm^−1^ corresponds to the phenolic hydroxyl O-H stretching vibration, and the peak at 2925.54 cm^−1^ corresponds to the C-H stretching vibration, representing functional groups within the persimmon tannin’s skeletal structure. These observations align with previous studies, affirming the abundance of phenolic hydroxyl groups in persimmon leaf tannins [50,51]. The peak at 1624.49 cm^−1^ represents the C=C stretching vibration, while the peak at 1727.22 cm^−1^ represents the C=O stretching vibration in carbonyl. Upon closer spectral examination, characteristic peaks of O-H and C=O stretching vibrations in the magnetic persimmon leaf composites underwent a blue shift to 3430.23 cm^−1^ and 1731.25 cm^−1^, respectively. This shift is possibly attributed to the formation and loading of Fe_3_O_4_ on the persimmon leaf surface, disrupting the intermolecular hydrogen bonding originally present in the persimmon leaves. This disruption led to a relatively weak blue shift of the hydroxyl and carbonyl peaks. Additionally, a characteristic peak of Fe-O is evident at 577.43 cm^−1^, confirming the successful loading of Fe_3_O_4_ onto the persimmon leaf surface. 

#### 2.1.2. X-ray Diffraction (XRD) Analysis

XRD analysis was conducted to further examine the structure of the prepared materials. The XRD patterns of the synthesized magnetic composites are illustrated in Figure 1B, showcasing diffraction peaks at 2θ angles of 29.9°, 35.8°, 42.38°, 52.1°, 56°, and 63.8°, corresponding to the (220), (311), (400), (422), (511), and (440) facets, respectively. These values align with the crystal planes of Fe_3_O_4_, as reported in previous studies [52]. This substantiates the successful preparation of the magnetic composites.

#### 2.1.3. Vibrating Sample Magnetometer (VSM) Analysis

VSM was used to test the magnetic properties of the magnetic composites. Figure 1C presents the hysteresis loop of the magnetic composites. As observed, the magnetic composites exhibit a hysteresis loop characteristic of superparamagnetism, and the magnetic saturation value reaches 12.8 emu·g^−1^. The experimental results indicate that magnetic composites can be rapidly separated from the solution under the influence of an external magnetic field, meeting the requirements for fast MSPE.

#### 2.1.4. Specific Surface Area and Pore Structure (BET) Analysis

The adsorption–desorption of N_2_ in the magnetic composites was tested to determine the specific surface area. As shown in Figure 1D the specific surface area of the magnetic composites increases with increasing pressure, accompanied by an increase in pore volume. The change in pore volume exhibits a slow increase when the relative pressure is below 0.9, followed by a rapid increase from 0.9 to 1. The range of 0.6–0.9 represents a hysteresis loop of pore volume change. The N_2_ adsorption–desorption isotherm of the magnetic composites can be classified as a Type IV isotherm. The presence of hysteresis indicates the presence of mesopores within the materials. Compared to the original persimmon leaves with a specific surface area of 1.3279 m^2^ g^−1^, the magnetic composite adsorbent has a specific surface area of 5.4688 m^2^ g^−1^, showing a significant increase that provides more adsorption sites.

#### 2.1.5. Scanning Electron Microscopy (SEM) Analysis

**Figure 2:**
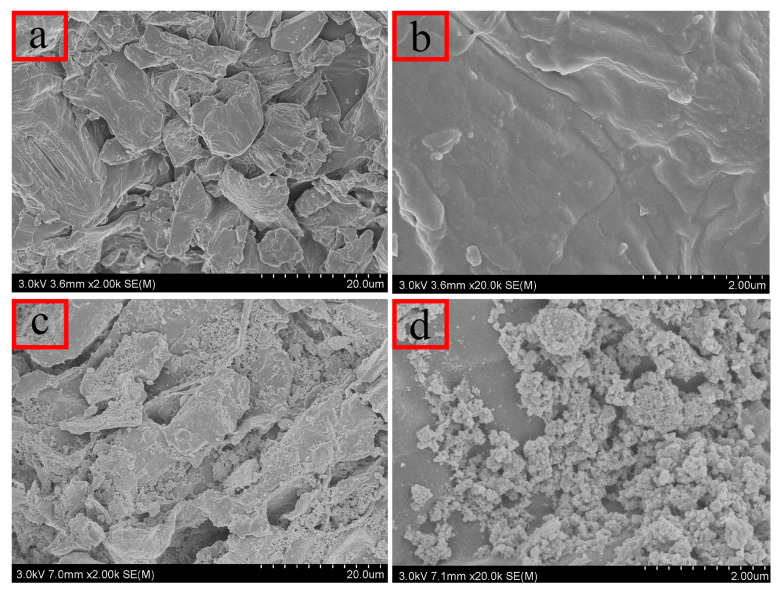
Scanning electron microscopy: (**a**,**b**) unmodified persimmon leaf particles; (**c**,**d**) magnetic persimmon leaf composites.

SEM was employed to examine the microstructure of the prepared materials. As illustrated in Figure 2a,b, untreated persimmon leaves display a layered structure with irregular surface features and discernible pores. These irregularities enhance the probability of contact between the material and analyte in the solution, facilitating adsorption. Figure 2c,d highlight numerous cracks and folds on the surface of the magnetic composites, providing additional adsorption sites and larger pores for analytes. Consequently, the increased specific surface area proves advantageous for effective adsorption.

### 2.2. Adsorbent Performance

**Figure 3:**
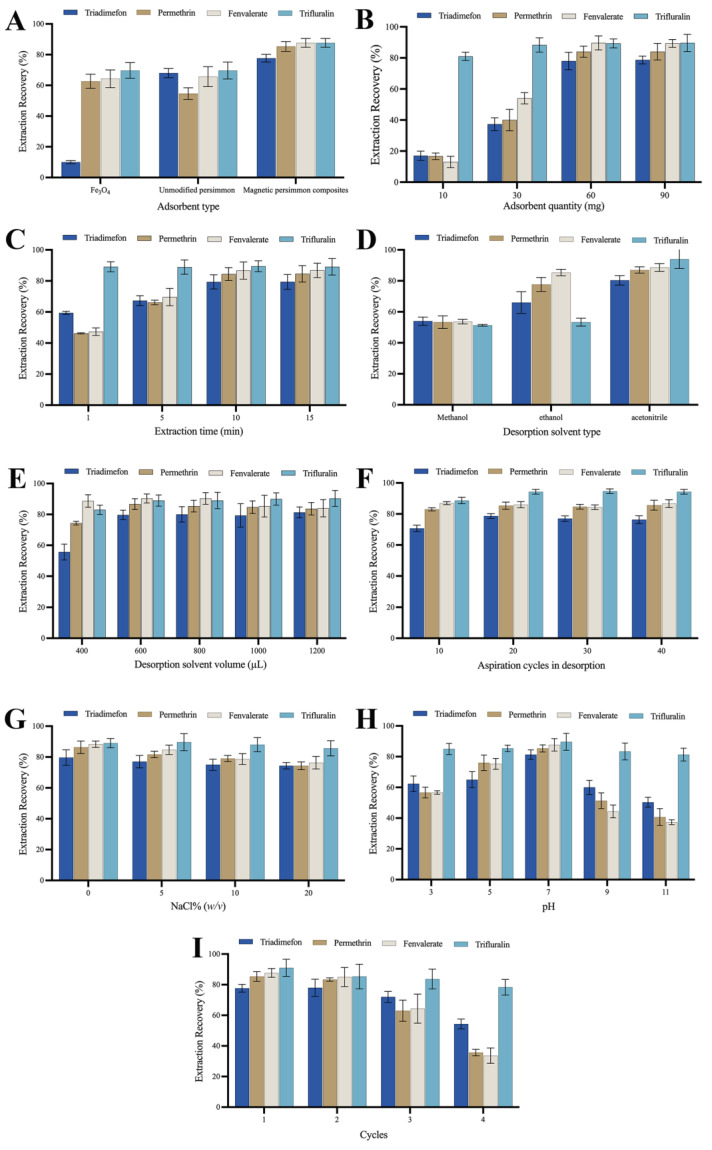
(**A**) Effect of the type of adsorbent; (**B**) Effect of the adsorbent amount; (**C**) Effect of the extraction time; (**D**) Effect of the desorption solvent type; (**E**) Effect of the desorption solvent volume; (**F**) Effect of aspiration cycles in desorption; (**G**) Effect of the ionic strength; (**H**) Effect of the pH; (**I**) Extraction reutilization of magnetic persimmon leaf composites.

To elucidate the adsorption performance of the prepared magnetic composites, this study conducted a comparative analysis of the extraction performance of 100-mesh unmodified persimmon leaf particles, magnetic persimmon leaf composites, and Fe_3_O_4_ nanoparticles synthesized via coprecipitation. Employing an equal quantity of these three adsorbents under consistent extraction conditions, the extraction recoveries for the target analytes were compared, as illustrated in Figure 3A. The results indicate that the magnetic persimmon leaf composites exhibited superior extraction performance compared to the other two materials. This superiority can be attributed to several factors: firstly, the presence of Fe_3_O_4_ nanoparticles increased the specific surface area and adsorption sites of the magnetic composites; secondly, the hydroxyl and carboxyl groups in persimmon leaves facilitated intermolecular hydrogen bonds with the target analytes, and the phenyl structure in persimmon leaves formed π-π interactions with the target analytes, thereby enhancing adsorption. Additionally, the experiments demonstrated that the magnetic persimmon leaf composites simplify the MSPE process. It offers the advantage of utilizing an external magnetic field for separation, resulting in improved extraction recoveries.

### 2.3. Study of Effective Parameters on Experimental 

#### 2.3.1. Adsorbent Amount

The quantity of adsorbent plays a pivotal role in achieving optimized adsorption performance. The adsorbent amount was investigated in the range of 10–90 mg to determine the optimized usage. Adsorbent amounts of 10, 30, 60, and 90 mg were tested. As depicted in Figure 3B, the recoveries of the four analytes exhibit a significant increase within the 10–60 mg range of adsorbent amounts. As the adsorbent amount increases, the recoveries gradually improve. This is likely because a higher amount of adsorbent provides more efficient adsorption sites for the target analytes, enhancing adsorption performance. Beyond 60 mg of adsorbent, the abundance of adsorption sites becomes excessive. To mitigate unnecessary usage, 60 mg was chosen as the optimized amount for magnetic persimmon leaf adsorbents in subsequent experiments.

#### 2.3.2. Extraction Time

MSPE relies on adsorption equilibrium. Appropriate extraction time ensures sufficient contact between the magnetic adsorbent and the analytes. Extraction time can affect the extraction performance of analytes before adsorption equilibrium is reached. To achieve higher extraction performance, extraction times of 1, 5, 10, and 15 min were tested. As shown in Figure 3C, with increasing extraction time from 1 to 10 min, the recoveries gradually improved. At an adsorption time of 10 min, the extraction recovery reached its maximum. Based on these results, an extraction time of 10 min was set.

#### 2.3.3. Desorption Solvent Type and Volume

Effective desorption solvent is a crucial component in the sample pretreatment process, and the choice of desorption solvent is key to improving extraction performance [53]. In this experiment, the influence of three organic solvents, namely methanol, ethanol, and acetonitrile, on desorption performance was compared. Figure 3D illustrates that acetonitrile provides the highest recovery compared to other desorption solvents. Consequently, acetonitrile was selected as the desorption solvent for subsequent experiments. The impact of acetonitrile volume (400 μL, 600 μL, 800 μL, 1000 μL, and 1200 μL) on the extraction performance of analytes was analyzed through optimization. As depicted in Figure 3E, the experimental results indicate that as the volume of acetonitrile increases from 400 μL to 600 μL, the recovery of all four pesticides gradually rises. When the desorption volume is set at 600 μL, the recoveries remain constant, achieving optimal desorption performance. Moreover, with a further increase in desorption volume, the recoveries of all pesticides do not significantly increase, and for some analytes, there is a slight decrease. Excess solvent not only leads to solvent wastage but also affects the enrichment effect. Therefore, 600 μL of acetonitrile was selected as the optimized desorption solvent in the subsequent experiments. 

#### 2.3.4. Aspiration Cycles in Desorption

Ensuring effective desorption is crucial for achieving higher extraction recoveries of analytes during the operation. Appropriate aspiration cycles allow analytes to be adequately desorbed from the adsorbent. However, excessive aspiration cycles not only fail to improve desorption efficiency but may also lead to the loss of desorption solvent, thereby reducing extraction recoveries. We systematically optimized the number of aspiration cycles—10, 20, 30, and 40 cycles—each lasting approximately 1.5 s. This corresponds to different desorption times of 15 s, 30 s, 45 s, and 60 s. The results are illustrated in Figure 3F. The experimental findings revealed that employing 20 aspiration cycles achieved the desired extraction recovery level. Further increasing the number of aspirations did not result in a significant change in extraction recoveries. Consequently, we selected 20 aspirations, equivalent to a desorption time of 30 s, for subsequent experiments. 

#### 2.3.5. Ionic Strength

The solubility of analytes in aqueous solutions may decrease with an increase in ionic strength, potentially leading to increased extraction performance. However, high ionic strength may reduce the diffusion rate of target analytes, resulting in decreased extraction performance. A slight decrease in the recoveries of the four analytes was observed as NaCl concentrations ranged from 0 to 20% (*w*/*v*). This might be attributed to the increase in sample solution viscosity with added salt concentration, reducing the rate at which analytes are adsorbed onto the adsorbent. Therefore, based on the results in Figure 3G, NaCl was not added in subsequent experiments.

#### 2.3.6. Sample Solution pH

The pH of the sample solution can influence both the existing form of analytes and the surface charge of the adsorbent. Therefore, the pH value of the sample solution was optimized to enhance extraction and recovery performance. The influence of pH values between 3.0 and 11.0 on the extraction performance of analytes was evaluated (see experimental results in Figure 3H). Within the pH range of 3.0–7.0, an increase in the sample solution’s pH led to an increase in recoveries. When the sample solution’s pH reached 7.0, the recoveries were maximized. Subsequently, the recoveries decreased as the sample solution’s pH continued to rise. A lower pH leads to a positively charged surface of Fe_3_O_4_, causing repulsion with the target analytes, and impeding effective adsorption. Extremely low pH may also compromise the stability of Fe_3_O_4_, thereby reducing its adsorption performance. As the pH increases and creates an alkaline environment, certain pyrethroid pesticides tend to decompose, leading to a significant reduction in their recoveries. For the other two analytes, trifluralin molecules feature two nitro groups and one trifluoromethyl group on the phenyl ring, all of which are strong electron-withdrawing groups. This configuration leads to easier dissociation of the two hydrogen atoms on the phenyl ring compared to when there are no strong electron-withdrawing groups. This results in the partial formation of trifluralin bases, exhibiting weak negative charges. Triadimenfon molecules undergo a similar process with the hydrogen on the triazole group dissociating under alkaline conditions, partially forming triadimenfon bases with weak negative charges. Meanwhile, the protons from the phenolic hydroxyl and carboxyl groups in persimmon leaves may dissociate, forming partial negative ions. This not only generates electrostatic repulsion but also diminishes π-π interactions between the phenyl structure in persimmon leaves and phenyl in pesticides. Additionally, alkaline conditions induce a negative charge on the Fe_3_O_4_ surface. The increased repulsive force between the adsorbent and weakly negatively charged analytes is unfavorable for adsorption. Consequently, as the pH increases, the adsorption performance of trifluralin and triadimenfon diminishes. Therefore, a pH of 7.0 was chosen as the optimum pH for the sample solution.

#### 2.3.7. Reusability

The number of reuse cycles is an important criterion for evaluating the stability and economic feasibility of magnetic persimmon leaf adsorbents. Choosing an adsorbent with good reusability can effectively reduce experimental costs. After each adsorption-desorption cycle, the magnetic persimmon leaf adsorbent was washed three times with ethanol and dried to ensure no residual analytes before the next MSPE process. As observed in Figure 3I, after two adsorption-desorption cycles, the adsorption performance did not significantly change. However, during the third and fourth cycles, a noticeable decrease in extraction recoveries was observed, possibly due to the loss of adsorption groups on the adsorbent’s surface. This suggests that the magnetic persimmon leaf adsorbent prepared in this study has good reusability for up to two cycles, demonstrating its environmentally friendly and cost-effective characteristics.

### 2.4. Adsorption Isotherms

**Figure 4:**
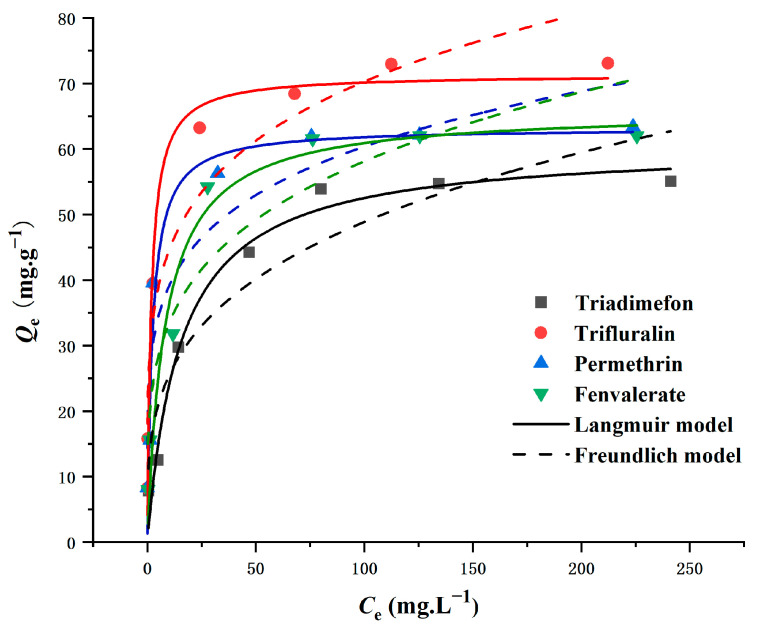
Adsorption isotherms of Langmuir and Freundlich for pesticide adsorption onto magnetic composites.

The interaction between magnetic persimmon leaf composites and four pesticides was investigated through adsorption isotherms. Adsorption experiments covered a range of initial pesticide concentrations from 10 to 300 mg L^−1^. As the initial concentration increased, the adsorption capacity of the magnetic persimmon leaf composites exhibited a rapid and equilibrating rise. This phenomenon is attributed to the elevated initial target concentration, which provides an enhanced driving force to overcome mass transfer resistance between solvent and solute surfaces [54]. The Langmuir model, suitable for simulating monolayer adsorption on surfaces with a limited number of similar sites, and the Freundlich model, designed for adsorption on amorphous surfaces assuming non-homogeneity, were employed [55,56]. The results, presented in Figure 4 and Table 1, reveal a well-fitting Langmuir isotherm with R^2^ values up to 0.97, indicating homogeneous adsorption of pesticides. The maximum adsorption capacities, calculated using the Langmuir isotherm model, were 73.75 mg g^−1^ for trifluralin, 58.07 mg g^−1^ for triadimefon, 65.35 mg g^−1^ for permethrin, and 63.82 mg g^−1^ for fenvalerate, closely aligning with actual data. Additionally, R_L_ values between 0 and 1, as per experimental results, suggest that the synthesized magnetic persimmon leaf adsorbent effectively facilitated the adsorption of the target analytes [57].

### 2.5. Method Validation

Under the optimized conditions, the linearity range, determination of coefficient, LOD, and LOQ of the method were evaluated. Table 2 lists the determination of coefficient and linear equations for the four analytes within a linear range of 0.75–1500 μg L^−1^. All determinations of coefficients were greater than 0.999. For the LOD, when the signal-to-noise ratio (S/N) of a chromatographic peak for a studied analyte reaches 3, the concentration corresponding to the peak area is defined as the LOD for that analyte. The LODs range from 0.25 to 1.1 μg L^−1^, and the LOQs range from 0.75 to 3.4 μg L^−1^ based on an S/N of 10. The LODs and LOQs were experimentally tested. In this experiment, the repeatability of the developed method for pesticide detection in environmental water samples was assessed through intra-day and inter-day precision. The intra-day RSD values range from 2.7% to 4.3%, and for inter-day precision, they range from 3.3% to 4.5%. This suggests that the method is feasible, reliable, and stable. 

### 2.6. Application of MSPE Based on Fe_3_O_4_/Persimmon Leaf Magnetic Composite to Real Water Samples

**Figure 5:**
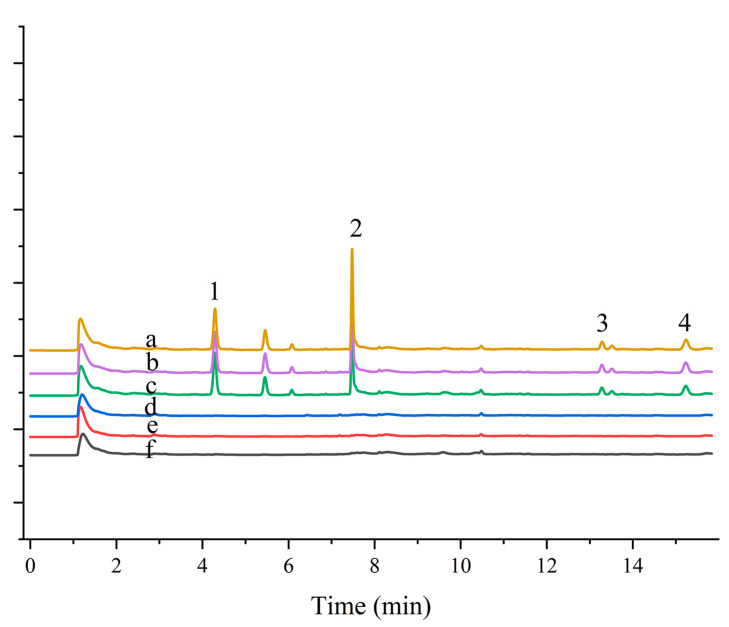
Application of magnetic persimmon leaf composite in environmental water sample analysis: (a) Pretreated Olympic Forest Park water sample spiked at 200 µg L^−1^; (b) Pretreated Beijing Sun Park water sample spiked at 200 µg L^−1^; (c) Pretreated Campus water sample spiked at 200 µg L^−1^; (d) Olympic Forest Park water sample not spiked; (e) Beijing Sun Park water sample water sample not spiked; (f) Campus water sample not spiked. Peaks: (1) Trifluralin (2) Triadimefon (3) Permethrin (4) Fenvalerate.

To further assess the applicability of the developed materials, magnetic composites combined with MSPE and GC-ECD were used to determine four pesticides in real water samples. The developed method was evaluated by sample recovery experiments on real samples at three different concentrations (20, 200, and 300 µg L^−1^). The results are presented in Table 3. Figure 5 shows the chromatograms of blank and spiked samples. The pesticides were not detected in lake water samples from Olympic Forest Park, Chaoyang Park, and Campus Linzhixin, and the relative recoveries of the spiked samples were between 80% and 95%, with RSD values ranging from 1.4% to 8.0%. This demonstrates the repeatability and reliability of the method for the determination of analytes in environmental water samples. 

### 2.7. Comparison of the Developed Method with Previously Reported Methods

The analytical performance of the established method was compared with other methods reported in the literature (Table 4). The results indicate that the proposed method offers a more satisfactory linear range, higher recoveries, and lower detection limits. MSPE avoids the need for centrifugation and filtration, making the extraction process simple. By using Fe_3_O_4_/persimmon leaf composite as the magnetic adsorbent, target analytes can be easily and rapidly separated from the sample solution, and the extraction time is only 10 min. The advantages of the magnetic persimmon leaf adsorbent used in this experiment include its simple synthesis, ease of operation, low toxicity, and cost-effectiveness. 

## 3. Materials and Methods

### 3.1. Reagents and Materials

Pesticide standards, including triadimefon, permethrin, fenvalerate, and trifluralin, and chemicals such as acetonitrile, polyethylene glycol, glutaraldehyde, ethanol, Fe_2_(SO_4_)_3_, and FeSO_4_.7H_2_O, were purchased from Aladdin Bio-Chem Technology Co., Ltd. (Shanghai, China). Sodium hydroxide (NaOH) was obtained from Modern Oriental (Beijing, China) Technology Development Co., Ltd. (Beijing, China), while methanol and NaCl were sourced from Macklin Biochemical Co., Ltd. (Shanghai, China).

### 3.2. Instrumentation

This study utilized a Nicolet iS10 FTIR spectrometer (Thermo Fisher, Waltham, MA, USA) to characterize the functional groups of unmodified persimmon leaf particles and magnetic persimmon leaf composites. Spectra were recorded within wave numbers ranging from 4000 to 400 cm^−1^. XRD analysis was conducted in continuous scanning mode on a D8 ADVANCE diffractometer (Bruker, Billerica, MA, USA) with an angular range between 10 and 90°. The scanning speed was set at 2° min^−1^. A HITACHI SU8020 scanning electron microscope (Hitachi, Tokyo, Japan) was employed to assess the morphological appearance of unmodified persimmon leaf particles and magnetic persimmon leaf composites. Samples were coated with gold to ensure electron conductivity, providing ample contrast in the SEM micrographs. Magnetic measurements were performed using a BKT-4500 vibrating sample magnetometer (Shincotest Technology, Beijing, China). Nitrogen adsorption–desorption isotherms were obtained through an ASAP 2460 micropore physisorption analyzer (Micrometrics, Norcross, GA, USA).

### 3.3. Preparation of Persimmon Leaf Particles

Persimmon leaves were collected on the BFU campus. The leaves were thoroughly cleaned using an ultrasonic cleaner. Subsequently, the cleaned persimmon leaves were dried in an oven at 105 °C for 8 h. The dried persimmon leaves were ground into powder using a milling machine and sieved progressively, finally passing through a 100-mesh sieve to obtain 100-mesh particles.

### 3.4. Preparation of Fe_3_O_4_/Persimmon Leaf Magnetic Composites

Fe_3_O_4_ nanoparticles were synthesized using an in situ coprecipitation method. After the formation of Fe_3_O_4_, they were simultaneously loaded onto persimmon leaf particles to prepare magnetic persimmon leaf composites. Initially, 8.0 g of FeSO_4_·7H_2_O and 5.56 g of Fe_2_(SO_4_)_3_ were weighed into a round-bottom flask and dissolved completely by adding 160 mL of ultrapure water with stirring. Next, 5 mL of polyethylene glycol aqueous solution was introduced as a surfactant into the flask with the mixed solution. Ten grams of persimmon leaf particles were added to the mixed solution. Subsequently, the mixed solution was heated to 70 °C and ultrasonicated for 30 min. Then, a prepared 0.50 mol·L^−1^ NaOH solution was added to the solution dropwise until the pH of the mixed solution reached above 9. The mixture was ultrasonicated continuously for two hours at 70 °C. The resulting powder was washed three times in ethanol and ultrapure water, respectively. Finally, the material was dried at 50 °C to obtain the Fe_3_O_4_/persimmon leaf magnetic composites.

### 3.5. Preparation of Standard Solution and Water Samples

Chromatography-grade acetonitrile was used to prepare pesticide standard stock solutions. Accurate amounts of four pesticide standards, including triadimefon, permethrin, fenvalerate, and trifluralin, were weighed in 10 mL bottles. Standard stock solutions of the pesticide were prepared at a concentration of 1000 mg L^−1^ and stored at 4 °C. A mixed standard solution was prepared by diluting the stock solutions with acetonitrile to achieve the desired concentrations. Real water samples were collected from various sources in Beijing (Olympic Park, Chaoyang Park, and BFU campus) to validate the proposed method. These water samples were collected in glass bottles, stored in a refrigerator at 4 °C, and protected from light. Before analysis, the water samples were filtered using a 0.45 μm microporous membrane to remove any suspended solids.

### 3.6. MSPE Procedure

A total of 60 mg of Fe_3_O_4_/persimmon leaf magnetic composite was added to a syringe containing 5 mL of the water sample. One end of the syringe was sealed with a stopper. The magnetic biomass adsorbent was dispersed by vortexing to ensure full contact with the analytes during a 10 min extraction. Subsequently, an external magnet was placed at one end of the syringe to collect the adsorbent. Following this, 600 μL of acetonitrile was added to the syringe for desorption, achieved by aspirating 20 times, taking approximately 30 s. The desorbed analytes were then collected with a microliter syringe. The desorption solvent was transferred to a vial for the GC-ECD analysis. Extraction recovery (ER) and relative recovery (RR) values were calculated using Equations (1) and (2), respectively. The calculation formulas are as follows:

ER = (C × V)/(C_0_ × V_0_) × 100% (1)

where C is the recovery concentration, V is the recovery volume, C_0_ is the spiked concentration, and V_0_ is the sample volume.

RR = (C − C_real_)/C_0_ × 100% (2)

where C is the recovery concentration, C_real_ is the concentration of analyte in the real sample, and C_0_ is the spiked concentration.

### 3.7. Chromatographic Conditions 

A GC-ECD fitted with a DB-5 (30 m × 0.25 mm × 0.25 μm) capillary column (Agilent 7890B, Agilent Technologies, Santa Clara, CA, USA) was used. The injection volume and temperature were 1 μL and 280 °C, respectively. The carrier and makeup gas was N_2_, and their flow rates were 3 mL min^−1^ and 17 mL min^−1^, respectively, with splitless injection mode. The detector temperature was kept at 280 °C. The initial oven temperature was 150 °C (held for 1 min). A series of ramping steps were performed as follows, the first ramp step was to 180 °C at 20 °C min^−1^ (held for 2 min), the second one to 230 °C at 25 °C min^−1^ (held for 5 min), the third one to 190 °C at 10 °C min^−1^ (held for 3 min), the fourth one to 230 °C at 25 °C min^−1^ (held for 1 min), and a final ramping step to 250 °C at 15 °C min^−1^ (held for 6 min).

## 4. Conclusions

In this research, we successfully prepared Fe_3_O_4_/persimmon leaf magnetic composites, employing them as a magnetic biomass adsorbent to establish an MSPE method for pesticide analysis in water samples. Comprehensive characterizations, including FTIR, XRD, VSM, surface area analysis, pore structure examination, and SEM, confirmed the successful synthesis of the magnetic composites and provided insights into their excellent adsorption performance. The optimized MSPE method coupled with GC-ECD demonstrated remarkable extraction recoveries ranging from 80% to 90%, using a minimal adsorbent amount (60 mg), and exhibited low LOD values within the range of 0.25–1.1 μg L^−1^. This technique, characterized by good stability, high sensitivity, accuracy, rapid separation, and reusability, stands as a valuable tool for environmental pesticide analysis. As a result, magnetic biomass composite adsorbents show great promise for environmental water sample analysis, contributing to the development of green and sustainable analytical methods. This study serves as a reference for the advancement and application of novel biomass composite materials in the field.

## Figures and Tables

**Table 1:** Adsorption isotherm parameters by using Langmuir and Freundlich models.

		Langmuir Model			Freundlich Model		
	Q_m_ (mg g^−1^)	K_L_ (L mg^−1^)	R_L_	R^2^	K_F_ (mg g^−1^ L^1/n^ mg^−1/n^)	1/n	R^2^
Trifluralin	73.75	0.4209	0.008–0.192	0.9780	21.00	0.279	0.9074
Triadimefon	58.07	0.0903	0.036–0.525	0.9742	9.42	0.362	0.8880
Permethrin	65.35	0.1346	0.024–0.427	0.9453	20.21	0.223	0.8643
Fenvalerate	63.82	0.1926	0.017–0.342	0.9480	13.46	0.329	0.8763

**Table 2:** Performance characteristics of magnetic persimmon leaf composites-based MSPE method combined with (GC-ECD).

Analyte	Linear Range (μg L^−1^)	Linear Equation	R^2^	LOD (μg L^−1^)	LOQ (μg L^−1^)	RSD (%) Intra-Day (*n* = 3)	RSD (%) Inter-Day (*n* = 6)	Extraction Recovery (%)
Trifluralin	0.75–100	y = 341.33 + 216.54	0.9994	0.25	0.75	3.1	4.3	90
Triadimefon	1.15–100	y = 414.38 + 192.73	0.999	0.38	1.15	4.3	4.2	80
Permethrin	3.4–1500	y = 79.979 + 223.08	0.9998	1.1	3.4	3.3	3.3	85
Fenvalerate	2.2–1500	y = 80.694 + 880.11	0.9996	0.73	2.2	2.7	4.5	87

**Table 3:** Relative recoveries of the studied pesticides in spiked water samples.

Analyte	Spiked Level (μg L^−1^)	Olympic Forest Park		Chaoyang Park		BFU Campus	
		RR (%)	RSD (%)	RR (%)	RSD (%)	RR (%)	RSD (%)
Trifluralin	20	82	4.1	82	1.4	84	2.3
	200	82	8.0	81	4.0	81	5.2
	300	91	3.1	89	2.8	86	3.7
Triadimefon	20	83	4.7	83	4.6	86	6.4
	200	80	2.0	80	3.2	81	2.3
	300	89	3.5	88	1.5	92	5.1
Permethrin	20	89	4.5	82	7.1	83	4.6
	200	93	5.3	93	3.4	95	3.1
	300	89	5.1	85	2.5	88	2.5
Fenvalerate	20	88	4.4	82	2.6	91	6.7
	200	93	2.8	93	2.8	94	2.2
	300	86	3.3	91	5.8	86	4.1

**Table 4:** Comparison between magnetic persimmon leaf composite-based magnetic solid-phase extraction and other analytical methods.

Method	Detection	Extraction Solvents	Sample	LOD (μg L^−1^)	Total Sample Preparation (min)	Extraction Recovery (%)	References
SPE ^a^	GC-MS	Carbon nanotubes	Olive oil	1.5–3.0	45	79–105	[58]
DSPE–DLLME ^b^	GC–FID	L-cysteine and sorbitol	Fruit juice	0.49–0.98	7	68-92	[59]
MSPE ^c^	GC-ECD	Carbon nanofibers	Water	1.44–5.15	16	70.0–120.6	[60]
MSPE	HLPC	Magnetic corn stalk biochar	Water and zucchini	0.03 (ng/g); 0.2–0.5 (ng/g)	20	86–113	[61]
MSPE	GC-ECD	Magnetic cork composites	Water	0.3–2.02	15	46–84	[44]
MSPE	GC-ECD	Magnetic persimmon leaf composites	Water	0.25–1.1	10	80–94	This work

^a^ Solid phase extraction (SPE); ^b^ Dispersive solid phase extraction-Dispersive liquid-liquid microextraction (DSPE-DLLME); ^c^ Magnetic solid-phase extraction (MSPE).

## Data Availability

The data that support the findings of this study are available from the corresponding author upon reasonable request.
